# Supplementary material for: Sacubitril/valsartan ameliorates cardiac function and ventricular remodeling in CHF rats via the inhibition of the tryptophan/kynurenine metabolism and inflammation
Source: Sci Rep. 2024 May 29;14:12377. doi: 10.1038/s41598-024-62472-7 (PMC11136956; doi:10.1038/s41598-024-62472-7)
Supplement: Supplementary file 1 — Supplementary Information 1. [file 41598_2024_62472_MOESM1_ESM.pdf]

Sacubitril/valsartan Ameliorates Cardiac Function and Ventricular Remodeling in CHF Rats via the Inhibition of the Tryptophan/Kynurenine Metabolism and inflammation

Authors: Jiali Gan[1†], Yuli Wang[1†], Yun Deng[1], Jiaqi Zhang[1], Shuangcui Wang[1], Xijuan Jiang[1], Maojuan Guo[1]*, Lili Song[2]*

Affiliations

[1]School of Integrative Medicine, Tianjin University of Traditional Chinese Medicine, Tianjin, China

[2]School of Chinese Materia Medica, Tianjin University of Traditional Chinese Medicine, Tianjin, China

[†]These authors have contributed equally to this work and share first authorship.

Abstract

Sacubitril/valsartan has been highly recognized as a treatment for Chronic Heart Failure (CHF). Its potential cardioprotective benefits and mechanisms, however, remain to be explored. Metabolomics can be used to identify the metabolic characteristics and related markers, as well as the influence of drugs, thereby opening up the new mechanism for sacubitril/valsartan therapy in CHF disease. In this study, the ligation of left anterior descending and exhaustive swimming were used to induce a rat model of CHF after myocardial infarction. The efficacy was appraised with echocardiography, serum NT-proBNP, and histopathologica. UPLC-Q/TOF-MS combined with multivariate statistical analysis approach were used to analyze the effect of sacubitril/valsartan on CHF rats. RT-qPCR and western blot were performed to investigate the tryptophan/kynurenine metabolism pathway. Accordingly, the basal cardiac functions were increased, while the serum NT-proBNP and collagen volume fraction decreased in CHF rats with sacubitril/valsartan.Sacubitril/valsartan regulated the expression of kynurenine et.al 8 metabolomic biomarkers in CHF rats serum, and it contributed to the cardioprotective effects through tryptophan metabolism pathway. In addition, the mRNA and protein expression of the indoleamine 2,3-dioxygenase(IDO)in the myocardial tissue of CHF rats, were down-regulated by sacubitril/valsartan, which was the same with the IL-1β, IFN-γ, TNF-α, COX-2, and IL-6 mRNA expression, and IL-1β, IFN-γ, and TNF-α expression in serum. In conclusion, sacubitril/valsartan can ameliorate cardiac function and ventricular remodeling in CHF rats, at least in part through inhibiting the tryptophan/kynurenine metabolism.

1 Introduction

Worldwide, Cardiovascular Diseases (CVDs) are the major cause of morbidity and mortality[1]. Chronic Heart Failure (CHF) is the culmination of an array of CVDs, and the high morbidity is still persistent[2]. Although significant efforts have been made to treat and manage CHF, mortality and hospitalization have continued almost unabated[3]. Therefore, the identification of novel therapeutic targets that improve the cardiac function in CHF remains a major priority.

As we known, the pathophysiology of CHF is characterized by activating both the Sympathetic Nervous System (SNS) and Natriuretic Peptides (NPs). Sacubitril/valsartan, recently, a neprilysin inhibitor and angiotensin receptor blocker, it has been confirmed that, can ameliorate cardiac function and myocardial remodeling[4], significantly decreases mortality and hospitalizations of CHF patients[5], and effectively prevents sudden cardiac death and progressive heart failure[6]. The latest guidelines for treating CHF in Europe and the USA support the use of sacubitril/valsartan[7]. In fact, CHF is a multifactor interaction network, in addition to the activation of neurohormonal systems, which is accompanied by immune inflammation and metabolism cross-talk[8]. Therefore, in addition to regulating neurohormonal systems, it is necessary to determine the other cardioprotective mechanisms of sacubitril/valsartan.

Metabolomics, which involves detecting metabolites in biofluids, cells, and tissues, is routinely used to find biomarkers. It can provide insight into the mechanisms that underlie disease processes and physiological conditions by detecting subtle changes in cellular pathways, widely used for the related research of cardiovascular disease[9].

The study of metabolomics has provided an insight into the metabolic changes that occurs in CHF, and it has been a means of identifying new CVDs biomarkers[10], which is helpful to clarify the metabolic perturbation of the medicine on the body and formulate personalized treatment strategies. Therefore, on the basis of exact protective effect of sacubitril/valsartan in CHF rats, we used UPLC-Q/TOF-MS to analyse the metabolic characteristics and inflammatory relationship and to clarify the new protective mechanism of sarcubitril/valsartan on CHF rats.

2 Materials and Methods

Animals, Chemicals, and Reagents

A total of 36, 7 ~ 8 week-old adult male Sprague-Dawley (SD) rats (body weight: 210 ~ 230 g) were provided by the Beijing vitalriver Laboratory Animal Technology (Beijing, China). The Ethics Committee for the Laboratory Animals at Tianjin University of Traditional Chinese Medicine approved the study. Sacubitril/valsartan was obtained from Beijing Novartis Pharma Ltd. (Beijing, China). Isoflurane was from Shanghai Yuyan Instruments Co.,Ltd. (Shanghai, China). The organic solvents used were obtained from Sigma-Aldrich (USA) and were of HPLC-grade.

Animals Models and Experiment Protocol

The rats were kept in a specific pathogen-free environment with a temperature of 20±2 °C, a relative humidity of 50%±5%, and a light/dark cycle of 12/12 hours. Free access to food and water was provided for animals. All rats were acclimatized for a week before the surgery. The rat model of post-myocardial infarction CHF was induced by left anterior descending (LAD) and exhaustive swimming[11]. Isoflurane inhalation anesthesia (4% induction concentration and 2% maintainance concentration, 2L/min)[12]. The LAD is sutured 1 ~ 2 mm below the left atrium using a needle holder, and then ligated. After ligation of the LAD, the left lower end will become pale, which indicated that the operation was successful. Sham group rats were only threaded without ligation. Thermal support was provided throughout the surgery procedure. Three days after surgery, all rats began an exhaustive swimming regimen. The water temperature shall be controlled at 21±1℃. In the exhaustive swimming tese, the rats sank into the water for more than 5 seconds and sank again after being pull out, which was repeated three times. Individual variations may dictate how many minutes each rat for exhaustive swimming test should last, but it should not surpass 30 minutes to ensure consistency and avoid any potential negative effects. The exhaustive swimming lasted for 4 weeks. The cardiac function of all rats was appraised with echocardiography. The left ventricular ejection fraction (LVEF) ≤45% meets the inclusion criteria. There were three groups, including sham, model, and sacubitril/valsartan (S) groups, n=9. In 2 ml of distilled water, 2.3 mg powder of sacubitril/valsartan was dissolved to make a concentration of 1.15 mg/ml drug solution. S group rats received 2 ml/d drug solution via oral gavage, sham and model groups rats respectively received equal volume of distilled water at the same time, which lasted for 4 weeks.

Echocardiography

Under isoflurane-induced general anesthesia, an echocardiography was performed via the VisualSonics Vevo 3100 (FUJIFILM VisualSonics, American) system. In order to record left ventricular activity, B-mode and M-mode ultrasound images were collected from MX201 (15MHz) ultrasound probes. The cardiac function parameters were recorded. The average value of each parameter was taken after three consecutive cardiac cycles. The LVEF and left ventricular diameter shortening rate (LVFS) were calculated by teichholz.

Histopathology Analysis of Cardiac Tissue

After measuring their body weights (BWs), all rats were euthanized by cervical dislocation, which meets animal ethics. Open the chest, and remove the whole heart. Histopathological assessment of cardiac tissue was performed on rats hearts fixed in 4% PFA for at least 72 hours. Myocardial infarction tissue below the ligation site was selected for cutting into slices. The histologic sections were stained with hematoxylin & eosin (H&E) and Masson trichrome and observed via an inverted microscope (Leica, Germany). The total area of collagen in the central muscle tissue of Masson section (expressed in pixel units) and the collagen volume fraction (CVF) of myocardium were measured and calculated by Image J image analysis software. CVF calculation formula: CVF = total area of myocardial collagen/total area of myocardium.

Enzyme linked immunosorbent assay

The serum was separated from the blood. The ELISA (Jiyinmei, Wuhan, China) were performed to determine the serum levels of IL-1β, IL-6, TNF-α, and N-terminal pro-B-type natriuretic protein (NT-proBNP). Operation steps followed manufacturers instructions.

Serum Sample Collection and Preparation

The whole blood was collected from the femoral artery after euthanasia. After centrifuging the serum for 10 minutes at 3,500 rpm at 4 °C, the serum was collected and stored at −80 °C for metabonomic analysis. Before UPLC-Q/TOF-MS, 100 µl of serum was mixed with 300 µl of cold acetonitrile and vortexed vigorously for 30 seconds. Injecting the supernatant into the instrument after centrifuging the mixture for 20 minutes at 4 °C, 13,000 rpm.

UPLC-Q/TOF-MS

In order to observe the metabolic effects after pharmacological intervention for CHF rats was undertaken with sacubitril/valsartan, UPLC-Q/TOF-MS, an untargeted metabolic analysis, was used. Separation was performed with the Waters ACQUITY HSS T3 system (2.1 mm × 100 mm, 1.8 µm). The mobile phase consisted of solvent A (0.1% formic acid–water) and solvent B (0.1% formic acid–acetonitrile). The conditions are as follows: flow rate of the mobile phase: 0.3 ml/minute, gradient elution: 0–0.5 minute, 99% A; 0.5-2 minutes, 99-50% A; 2–10 minutes, 50–1% A; 10.5-15 minutes, 1-99%A, column temperature: 45±1℃, injection volume: 5 µl. MS analysis was performed on a hybrid quadrupole-time-of-flight (Q-TOF) mass spectrometer (Wates Xevo G2 Q-TOF MS systems, USA), and equipped with an electrospray ionization (ESI) source. Ion source temperature of 120℃, desolvation temperature of 300℃, cone gas flow of 50 L/h, and desolvation gas flow of 300 L/h, capillary voltage of 1.0 kV, the range of relative molecular mass is 50-1200*m/z*. Mass spectra was obtained in positive and negative ion modes, respectively. The data was collected and processed by Masslynx V4.1.

Multivariate Statistical Analysis

The raw data was exported from Masslynx v4.1 workstation. For the purpose of normalizing the absolute concentration of metabolites, the data was corrected by peak matching, peak alignment, and peak area normalization. Retention time (tR)-*m/z* datasets were used to characterize the detected ions. In the SIMCA-P 14.1 software package, each tR-m/z variable's intensity was normalized and imported for principal component analysis (PCA) and partial leastsquares-discriminant analysis (PLS-DA). PCA was used to evaluate the clustering characteristics of Sham, Model and S groups, and PLS-DA can reflect the differences between the groups to the greatest extent. Based on the potential biomarkers of CHF rats, the relative contents of metabolic markers were compared. The metabolic markers with callback trend were extracted as potential targets with sacubitril/valsartan treatment, which were enriched into metabolic pathways via the MetaboAnalyst 5.0 website.

Real-time quantitative Polymerase Chain Reaction

Following the manufacturer's instructions, TriQuick reagent (Solarbio, China) was used to extracte the total RNA from myocardial tissues of left ventricular anterior wall. The reverse transcription kit (TIANGEN, China) was used to produce cDNA from 1 µg total RNA. Ultra SYBR color qPCR Master Mix (Comwin, China) was used for real-time quantitative polymerase chain reaction analysis. The housekeeping gene GAPDH was used to normalize the expression of the relative genes. The relative expression of the sample gene was calculated by dividing the target gene by the reference gene using the equations: ΔCt (sample) = Ct (target) – Ct (reference); relative quantity = $2^{-\Delta Ct}$. The experiments were repeated triplicatly, n = 5.

Western Blotting Analysis

A phosphatase inhibitor (Solarbio) was used to extract proteins from cardiac tissues using RIPA lysis buffer (Solarbio, Beijing, China). The BCA protein assay (Thermo Fisher Scientific, American) was used to determine soluble protein concentrations. SDS-PAGE gel (Solarbio) was used to separate 20 µg proteins. Polyvinylidene difluoride membranes (Millipore, American) contained the protein bands. The western blots were cut prior to hybridisation with the primary antibody during the imprinting process. The primary antibody, IDO (sc-53978, Santa Cruz, American), was incubated overnight at 4°C after being blocked for 4 hours with blocking buffer (5% skimmed milk, 150 mM NaCl, 20 mM Tris HCl, 0.05% Tween-20, and pH 7.6). Incubate the secondary antibodies (ABCAM), labeled with horseradish peroxidase, for 2 hours at room temperature. Image J was used to analyze the densitometric differences between the bands, when they were visualized by chemiluminescence (ECL, Millipore).

Statistics Analysis

The data was analyzed by IBM SPSS 21.0 software, and was shown as means ± standard deviation when was under normal distribution, and the one-way ANOVA was used to compare the differences between groups. The LSD test was used when the variance was uniform; while the variance was not uniform, Dunnett' T3 test was used to compare

the difference. The difference was statistically significant when *P*<0.05 was used. The statistical graphs were drawn by Graphpad prism 7.

3 Results

3.1 Sacubitril/valsartan Ameliorates cardiac function and left ventricular remodeling in CHF rats

Our experiment results indicate that the rat model of post-MI CHF was established by ligation of the LAD and exhaustive swimming (**Fig.S.1 and Tab.S.1**). After 4 weeks of sacubitril/valsartan treatment, echocardiography was performed to assess cardiac function. Compared with CHF model group, sacubitril/valsartan elevated significantly LVEF and LVFS (P<0.05), and reduced significantly LVID; S (P<0.05) **(Fig.1a and Tab.S.2)**. NT-proBNP was remarkably decreased with sacubitril/valsartan(S) group versus CHF model group (P<0.01) **(Fig.1b and Tab.S.2)**. The general view showed that the left ventricle of the heart of CHF rats was gray white and inelastic with sclerosed tissue attached to it. The heart volume of sacubitril/valsartan group was smaller versus CHF model group, and the area of gray white and inelastic scar-tissue on the front wall of left ventricle was reduced **(Fig. 1c)**. HE and Masson staining of the cardiac tissue section also confirmed that sacubitril/valsartan significantly attenuated cardiac injury and fibrosis **(Fig. 1d and 1e)**. Furthermore, Masson staining showed that sacubitril/valsartan substantially reduced collagen volume fraction (CVF) (P<0.05) **(Fig.1f and Tab.S.2).** Together, these results suggest that sacubitril/valsartan inhibits left ventricular remodeling,attenuates cardiac fibrosis, and ameliorate systolic and diastolic function in CHF rats.

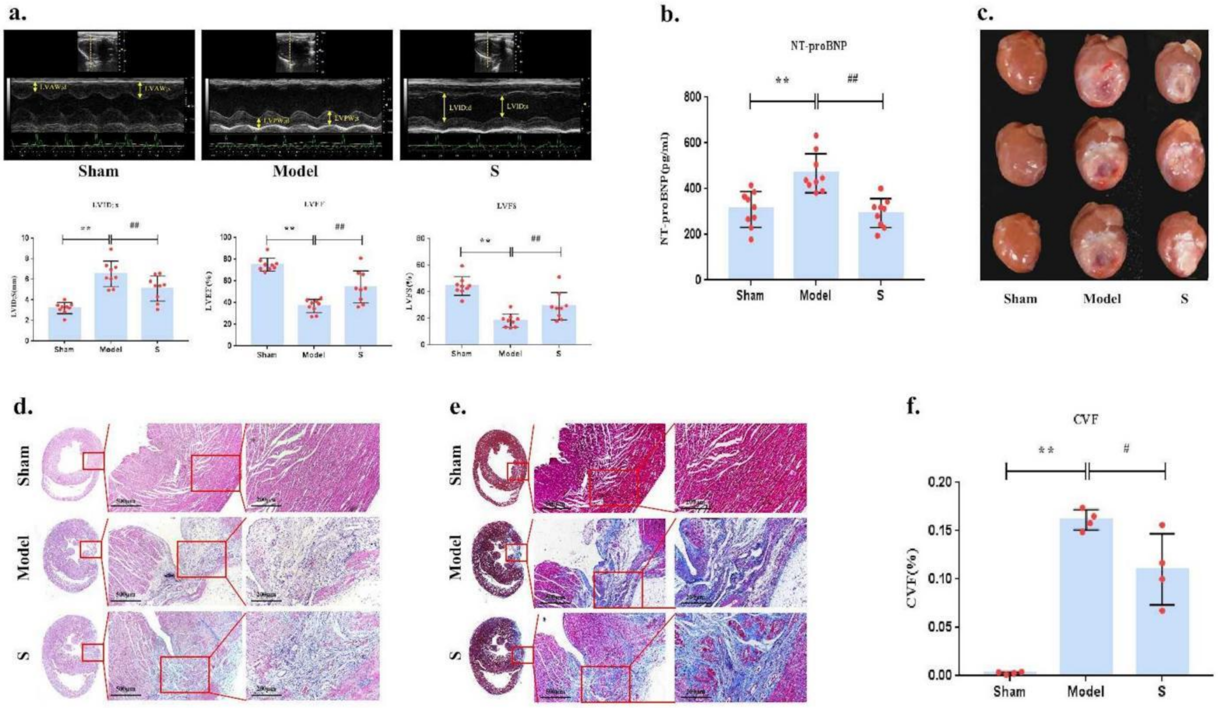

Figure 1 Sacubitril/valsartan inhibits left ventricular remodeling and Ameliorates cardiac function in CHF rats

(a) M-mode echocardiographyand cardiac function parameters. LVID;s, Left ventricular internal diameter of systole; LVEF, Left ventricular ejection fraction; LVFS, Left ventricular fractional shortening. **(b)** NT-proBNP in serum. **(c)** General view of heart. **(d)** HE stain image. **(e)** Masson stain image. **(f)** CVF, Collagen volume fraction. Compaerd with Sham group, $^{**}P < 0.01$, compared witah CHF model group, $^{\#\#}P < 0.01$, $^{\#}P < 0.05$.

3.2 Serum Untargeted Metabolic Profiles Analysis

3.2.1 Effect of Sacubitril/valsartan on Serum Metabolic Profile in CHF Rats

After four weeks with sacubitril/valsartan, different serum metabolites were analysed in CHF rats in order to shed further light on metabolic profiling. Based on rats serum chromatogram with base peak intensity (BPI), ESI positive (ESI+) and negative (ESI-) were measured. As is shown in **Figure 2**, the (tR)-*m/z* of the chromatographic peaks is basically consistent, the baseline is stable, and the separation is good.

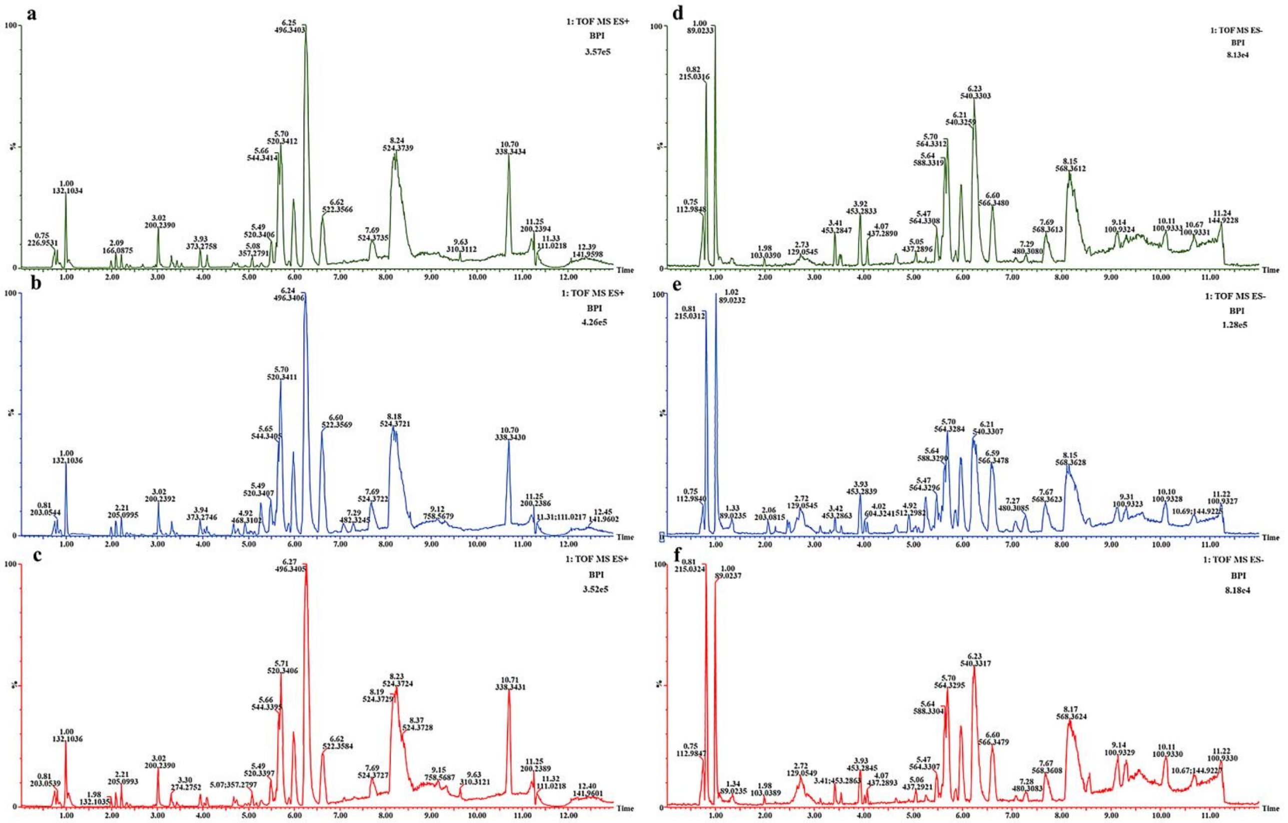

Figure 2 BPI of the rat serum in the ESI positive (ESI+) and negative (ESI-) mode.

(a) BPI in ESI+ mode of Sham gruop. **(b)** BPI in ESI+ mode of Model gruop; **(c)** BPI in ESI+ mode of S gruop. **(d)** BPI in ESI- mode of Sham gruop. **(e)** BPI in ESI- mode of Model gruop. **(f)** BPI in ESI- mode of S gruop.

Multivariate statistical analysis was carried out with SIMCA-P software. An unsupervised PCA demonstrates that, compared to the sham group, the CHF model group displayed different metabolic profiles. With the OPLS-DA, a supervised pattern recognition technology, metabolic changes in the CHF model group were compared with those in the sham group, which could aid in biomarker discovery. According to the S-plots, there was a significantly greater separation between the sham group and the CHF model group due to the ions farther from the origin, and the ions may be considered the distinguishing metabolites of CHF rats. The S-plots, the VIP value (VIP>1) from the OPLS-DA analysis, and the t-test (P<0.05) could then be combined to identify the corresponding metabolites. We have been reported that 22 metabolic markers of CHF rats in the journal of Prostaglandins Other Lipid Mediat (Wang S et al., Shengmai Yin formula exerts cardioprotective effects on rats with chronic heart failure via regulating Linoleic Acid metabolism. Prostaglandins Other Lipid Mediat. 2022 Feb;158:106608.doi: 10.1016/j.prostaglandins.2021.106608.).

In order to observe the effect of sacubitril/valsartan on serum metabolism in CHF rats, the data from the Sham, Model, and S groups were analysed by the PCA and the PLS-DA. The positive mode parameters are R^2Y=0.859, Q^2=0.433, and the negative mode parameters are R^2Y=0.889, Q^2=0.538, indicating that the interpretation rate and prediction ability of the three groups of PLS-DA models are also good (**Tab. S.3**). The PCA shows that they have a certain discrete trend. The metabolic trend of CHF model group rats is far away from the sham group, and the sacubitril/valsartan group rats tend to be close to the sham group (**Fig. 3**), which indicates that sacubitril/valsartan can interfere with the metabolism of CHF rats, and make them becoming more metabolically normal.

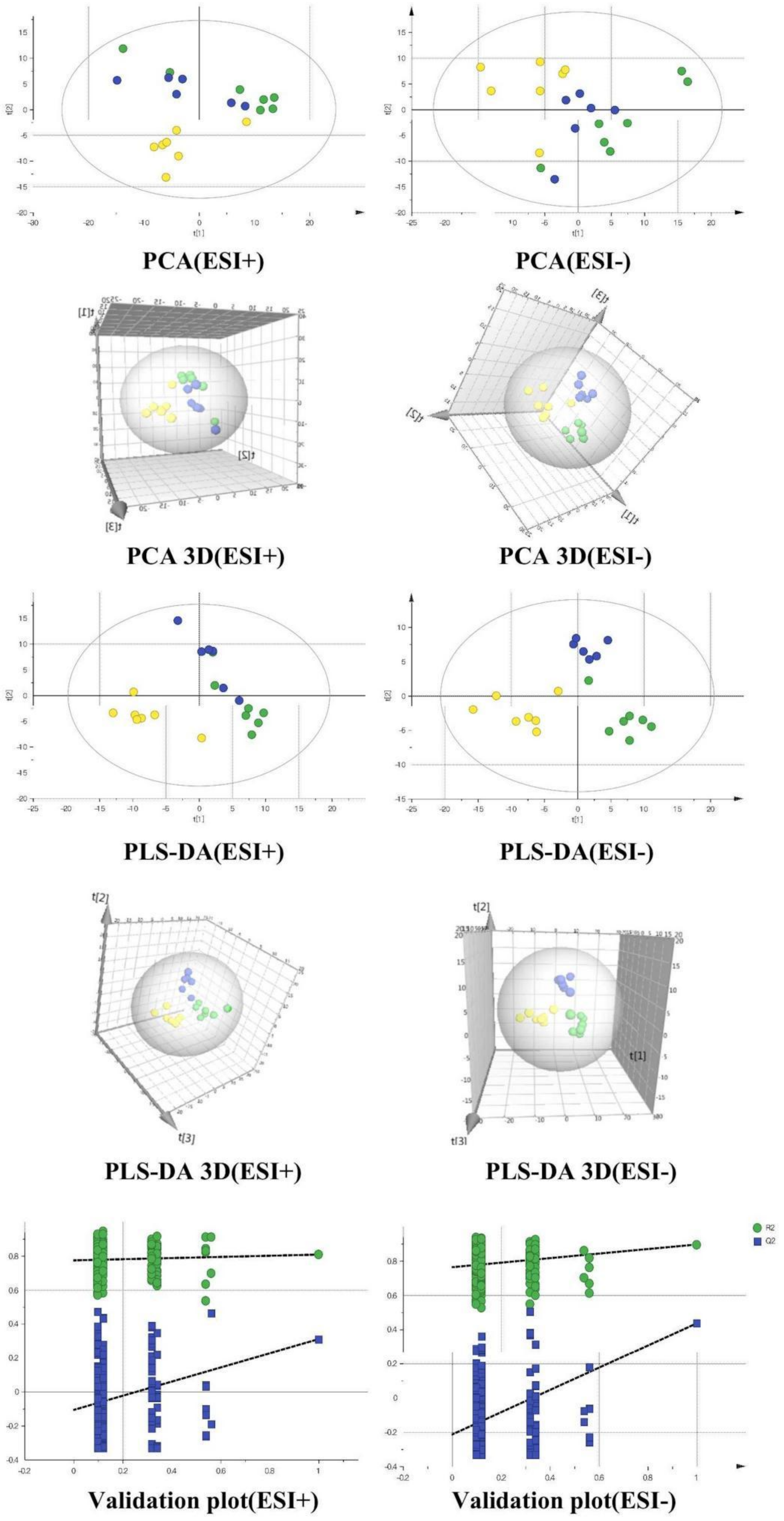

Figure 3 Serum Metabolic Profiles Analysis

PCA, PCA 3D, PLS-DA and PLS-DA 3D scores plot, validation plot from PLS-DA, sham group (green), CHF model group (blue) vs. sacubitril/valsartan(S) gruop (yellow).

3.2.2 Metabolic Pathway Analysis of Sacubitril/valsartan for the treatment of CHF Rats

Compared the relative contents of 22 metabolic markers, 8 metabolic markers exhibited a callback effect in the sacubitril/valsartan group, including 8-Hydroxyguanine, Cervonoyl ethanolamide, LysoPC(22:6), β-Guanidinopropionic acid, Kynurenine, Tryptophan, Proline betaine, and Threonine **(Fig. 4a)**. The metabolic pathway enrichment analysis and metabolite topology analysis of identified metabolites were through the MetaboAnalyst 5.0 website (https://www.metaboanalyst.ca). An overview of pathway analysis is shown in **Fig.4b** and **Tab.S.4**. All pathways matched in the pathway analysis are included. According to pathway enrichment analysis, those pathways are arranged by *p*-value on the Y-axis, and pathway impact values are on the X-axis from pathway topology analysis. In addition, according to the *p*-value, the node color is graded, and the node size is in line with the path influence value. Therefore, many metabolic pathways including tryptophan metabolism, glycine, serine and threonine metabolism, glycerophospholipid metabolism, valine, leucine and isoleucine biosynthesis, and aminoacyl-tRNA biosynthesis are significantly affected. Among them, the tryptophan metabolism is most significant, -log(p)=1.7402, impact=0.23722. These findings suggest that sacubitril/valsartan for the treatment of CHF rats is related with tryptophan metabolism, its metabolic pathway diagram is shown in **Fig.4c**. It is exciting, kynurenine and tryptophan belong to tryptophan metabolism pathway.

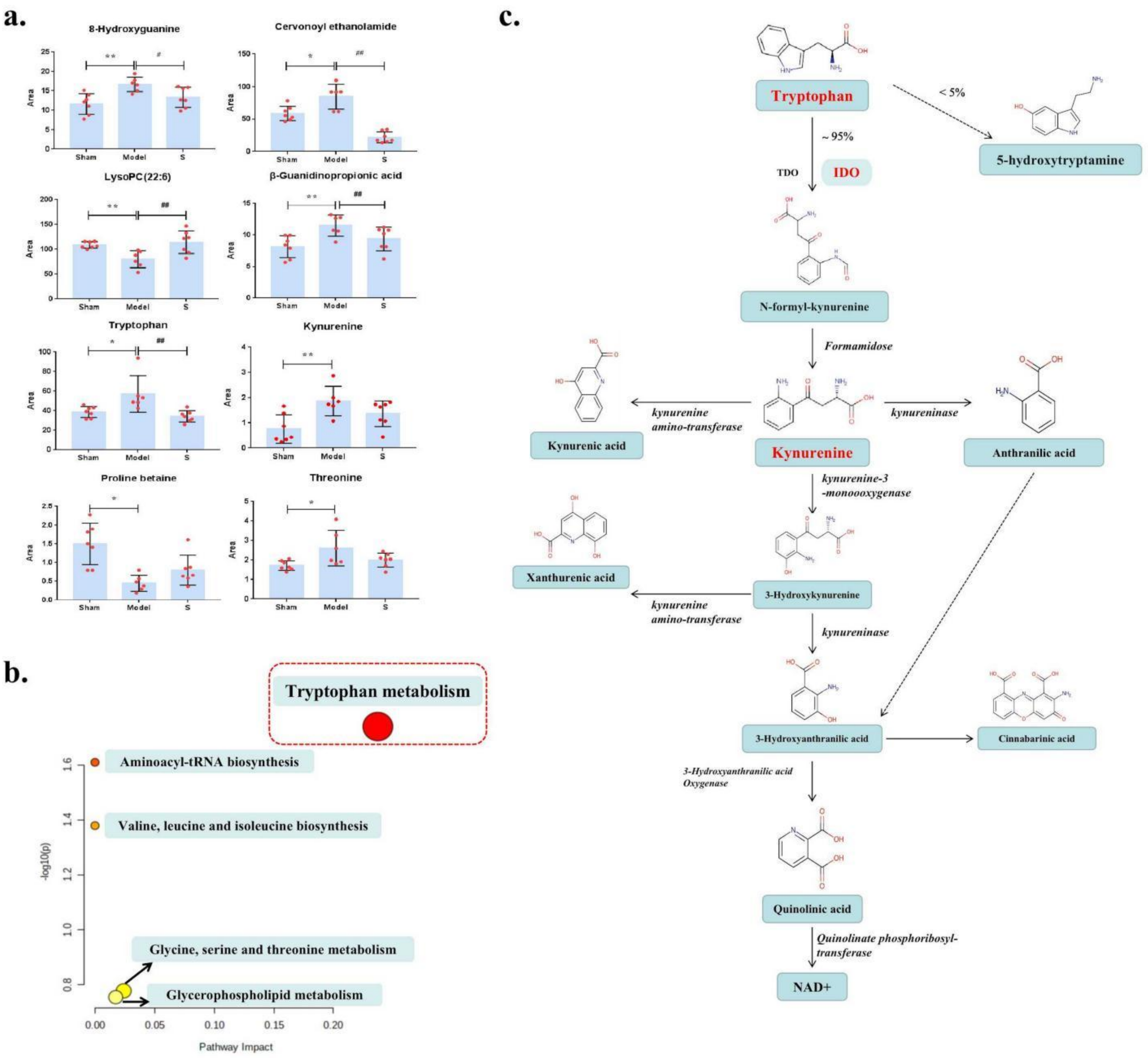

Figure 4 Metabolic Pathway Analysis of sacubitril/valsartan for the treatment of CHF Rats

(a) Relative levels of 8 metabolic markers. Compared with Sham group, $^{*}P<0.01$, $^{**}P<0.05$; Compared with CHF model group, $^{\#\#}P<0.01$, $^{\#}P<0.05$. **(b)** Metabolic Pathway Analysis Map. **(c)** Schematic diagram of the Tryptophan pathway. Over 95% of tryptophan are metabolized to kynurenine via indoleamine-2,3-dioxygenase (IDO) or tryptophan-2,3-dioxygenase (TDO), and several metabolites are generated downstream of the pathway. The

remaining fraction of tryptophan (< 5%) are used for protein synthesis, and the production of neurotransmitters (serotonin) and neuromodulators (tryptamine).

3.3 Effects of Sacubitril/Valsartan on IDO and Its Related Inflammation Inducing Factors

IDO is the first key rate-limiting enzyme in the tryptophan metabolic pathway, which promotes tryptophan catabolizing into kynurenine. The results show that, compared with the CHF model group, the mRNA and protein levels of IDO in sacubitril/valsartan (S) group were significantly downregulated (*P*<0.05) **(Fig.5a and 5b)**. It is suggested that sacubitril/valsartan inhibits the IDO of myocardial tissue in CHF rats.

The inflammatory factors induce the activation of IDO， such as IFN-γ, TNF-α, IL-1β, IL-6 and COX-2, our results also surpport it. Compared with the Sham group, the mRNA levels of IL-1β, IFN-γ, TNF-α, IL-6, and COX-2 were significantly increased in the CHF model group (*P*<0.01). The mRNA expression levels of the above inflammatory factors were significantly downregulated after sacubitril/valsartan treatment (*P*<0.01, *P*<0.05) **(Fig.5c)**. In addition, sacubitril/valsartan significantly decreased the serum levels of IL-1β, IFN-γ, and TNF-α in CHF rats (*P*<0.01, *P*<0.05) **(Fig.5d)**. These results show that the expression of inflammatory factors in serum and myocardial tissue mRNA levels are consistent trend, which indicates that sacubitril/valsartan can attenuate the inflammatory response in CHF rats.

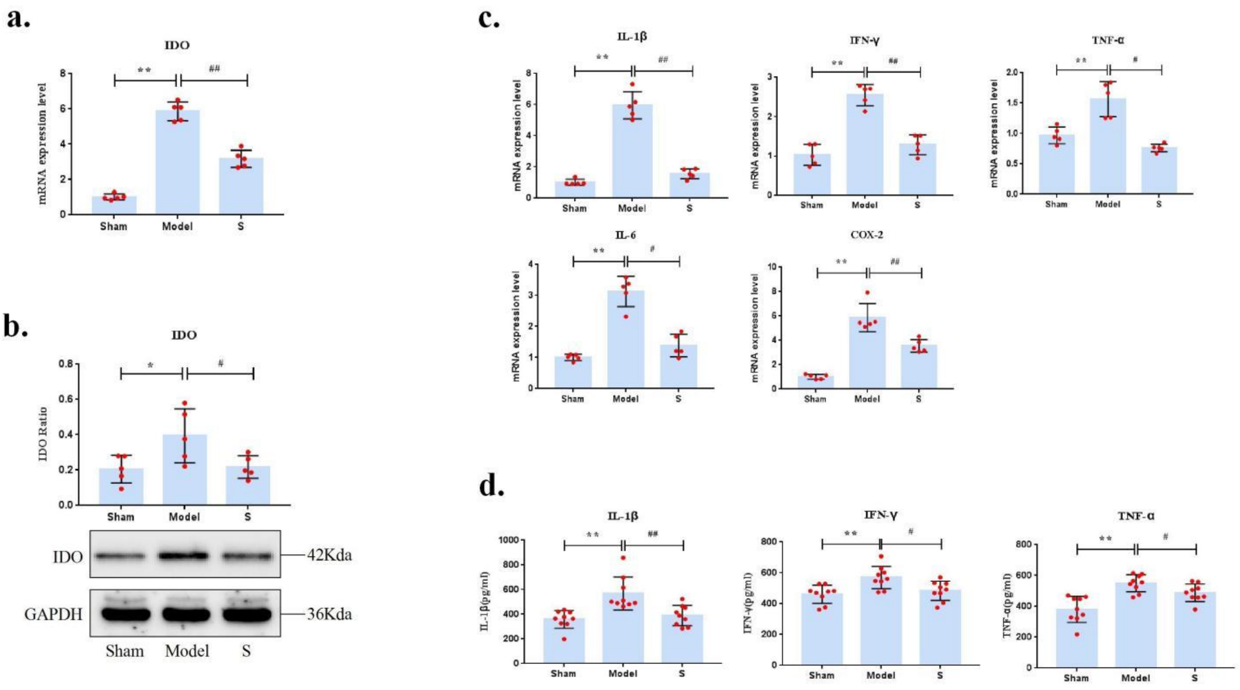

Figure 5 Effects of sacubitril/valsartan on IDO and Its Related Inflammation Inducing Factors

(a) The mRNA levels of IDO in myocardial tissue. **(b)**The protein level of IDO in myocardial tissue. **(c)**The mRNA levels of inflammatory factors in myocardial tissue. **(d)** The level of inflammatory factors in serum. Compared with sham group, $^{**}P$<0.01, $^{**}P$<0.05, compared with model group, $^{\#\#}P$<0.01, $^{\#}P$<0.05.

4 Discussion

Chronic Heart failure(CHF) involves an insufficient supply of oxygen to tissues and organs, as well as a decrease in cardiac output. It is often accompanied by cardiac dysfunction and insufficient ejection fraction clinically. CHF is a complex condition, and no single indicator can adequately describe all of the changes that occur as a result[13]. In this study, we used both echocardiography and NT-proBNP to characterize cardiac functions, and histopathology is used to assess the area of myocardial infarction (MI) size and myocardial fibrosis. LVEF≤45% is inclusion criteria, a significant reduction in the overall cardiac function in CHF model rats. Moreover, NT-proBNP, as a response to wall stress, is secreted the ventricular myocardium, and it is a widely used biomarker, diagnosis and prognosis of heart failure[14]. In CHF model rats, NT-proBNP is above the normal range , which is consistent with the fact. The scar area was calculated by using the morphological and morphometric data. In the CHF model rats,

myocardial infarction and fibrosis were significant. It is clear that this is a true animal model of CHF, despite the absence of descricbtions about expiratory dyspnea, lung rales, or beat rhythms.

Sacubitril/valsartan is a widely used medication for the treatment of heart failure and is commonly referred to as a new generation ARNI (angiotensin receptor-neprilysin inhibitor)[15]. There are two components in the drug , valsartan and sacubitril. As an angiotensin II receptor antagonist, valsartan can reduce cardiac workload and improve the function of the heart by blocking the detrimental effects of angiotensin II on the heart and blood vessels[16]. Sacubitril, on the other hand, a neutral endopeptidase inhibitor, prevents the breakdown of natriuretic peptides, which increases in their levels and ultimately provides a cardioprotective effect[17]. Many researchs reinforce the robust clinical benefits of sacubitril/valsartan in CHF[18]. Our results suggest that sacubitril/valsartan has a protective effect on CHF rats. The findings of other studies, support the key role that sacubitril/valsartan plays in the reduction of collagen deposition in infarcted areas, as well as the development of compensatory hypertrophy in non-infarcted areas, and eventually improves cardiac structure and function in myocardial infarction rats[19, 20]. The strong role of sacubitril/valsartan in the treatment of other diseases has also been confirmed. There are some data on the beneficial effects of sacubitril/valsartan for a patient's diabetes mellitus status, irrespective of haemoglobin (Hb) A1c concentration[21]. It is also beneficial for renal function, by reducing the serum creatinine[22], and for Alzheimer's disease, by interfering with amyloid β homeostasis[23]. In addition, there is preclinical evidence indicating that sacubitril/valsartan has a significant improvement in doxorubicin-induced cardiotoxic heart failure. Specifically, sacubitril/valsartan reduces the levels of TNF-α, IL-1β, IL-6, and NT-proBNP in doxorubicin-induced cardiotoxic mice[24]. In another clinical case report, sacubitril/valsartan was shown to effectively treat doxorubicin-induced cardiomyopathy[25]. Similarly, empagliflozin, an inhibitor of sodium-glucose cotransporter 2 (SGLT-2), showed cardioprotective effects in mice exposed to doxorubicin-induced cardiotoxicity. It is done by regulating NLRP3 and MyD88-related pathways, reducing inflammation and fibrosis[26]. It is also important to note that empagliflozin reduces hospitalization and cardiovascular deaths among patients with heart failure[27]. These findings suggest new therapeutic options for patients with anthracycline-induced heart failure.

Due to the complexity of the drug itself and the endogenous environment of the body, in spite of the obvious biological plausibility of the effects sacubitril/valsartan's effects on NPs and RAAS in CHF, it cannot be explained fully how it is able to exert such beneficial clinical effects. Therefore, it is necessary to conduct further studies, from multiple perspectives, to explain protective the effects of sacubitril/valsartan. It has been revealed that CHF is universally linked to an abnormal metabolism, regardless of the etiology and symptoms[28]. Therefore, keeping the metabolic regulation stable is a new treatment of CHF. However, there are only a few studies on sacubitril/valsartan, from a metabolomics perspective. In this study, we studied UPLC-Q/TOF-MS metabolic profiles of CHF rats and identified the metabolic pathways associated with sacubitril/valsartan in rats with CHF. Finally, we used molecular biology techniques to give a preliminary insight into the relationship between sacubitril/valsartan interference in the metabolism and inflammation of CHF rats.

Impaired tryptophan metabolism occurs in CHF, although the underlying mechanisms remain unclear. IDO is the first rate-limiting enzymes of the degradation of kynurenine by tryptophan in extrahepatic tissues[29]. There are two isoforms of IDO, IDO1 and IDO2, of which IDO1 is the major one controls tryptophan degradation[30]. When the immune system is activated, for example, inflammation, it can enhance the kynurenine pathway by triggering rate-limiting enzymes IDO[31]. It has been reported IDO is a marker for significant coronary artery disease, its activation correlated with the extent and severity of disease[32]. A Mendelian Randomization study had reported that IDO1 might be a potential therapeutic target for ischemic heart disease , which means IDO1 also plays an important role in heart disease in humans[33]. According to reports, IFN-stimulated response elements (ISREs) and IFN-activated sites (GASs) are found in the promoters of the IDO gene in mammals[34], so IFN-γ is a strong inducer of IDO[35]. In addition, other cytokines (TNF-α, IL-1β, and IL-6) are also able to induce IDO activation, although to a much lesser extent[35]. Surprisingly, quinolinic acid, the downstream product of the tryptophan metabolism pathway, could promote the synthesis of inducible nitric oxide synthase (iNOS)[36]. Under the action of iNOS, arginine produced less NO and more superoxide radical (O^{2-}). O^{2-} and 3-Hydroxyanthranilic acid enhance lipid peroxidation and promote arachidonic acid to produce prostaglandin E (PGE) and leukotrienes (LT) under the action of COX-2 and 5-LOX, respectively, which triggers an inflammatory response. It indicates that there is cross-talk between Trp/Kyn metabolism and inflammatory response. Schiattarella GG suggests that metabolic disturbances and inflammation are prevalent in HFpEF[8], while Li Z et al. propose that an important role is played by metabolic remodeling and chronic inflammation in CHF and its complications[37]. It is therefore traceable.

As confirmed, NLRP3 expression levels increase in the early stages of ventricular remodeling after myocardial infarction[38]. Subsequently, NLRP3 combines with a caspase-recruitment domain (ASC) to recruit and activate caspase-1, promoting the maturation and release of IL-1β[39]. The release of IL-1β recruits inflammatory cells and mediators to the site of myocardial injury, such as IL-6 and TNF-α, which initiates and amplifies the inflammatory process and accelerating myocardial cell death, ultimately leading to adverse remodeling of the ventricular tissue and the development of heart failure[40]. In this study, the elevation of the inflammation markers, such as IL-1β, INF-γ, and TNF-α, suggests that the CHF model is highly immune-activated. There is still a visible inflammatory state present in CHF even though it is in later period. It may be due to the interaction between the RAAS and immune activation, the increase in monocytes, as well as matrix metalloproteinases stimulating in the generation of pro-inflammatory collagen[41]. Fortunately, sacubitril/valsartan can inhibit the accumulation of ROS decrease NLRP3 expression[42]. Also, it can downregulate the expression levels of pro-caspase-l, pro-IL-lβ, and pro-IL-18, and inhibit the release of IL-lβ and IL-18[42]. Consistently, in this study, sacubitril/valsartan was found to markedly decreased the levels of inflammatory factors IL-1β, INF-γ, and TNF-α in rats with CHF model. It is due to its inhibition of Ang II type 1 receptor activity, inhibition of bradykinin degradation by neprilysin, and inhibition of the inflammatory response. Even studies suggest that sacubitril-valsartan can reduce inflammation and fibrosis in the heart by directly decreasing the production and release of IL-1β[43].

In fact, the CHF model actually raises serum tryptophan by 45% and kynurenine by 125% in this study. During CHF, stress enhances catecholamine levels, which may lead to the release of fatty acids-bound tryptophan from serum albumin[44]. When tryptophan oxidation exceeds renal handling, such as after robust induction of TDO and IDO or loading with tryptophan or kynurenine, serum kynurenine can increase[45]. The greatly elevated kynurenine in the present study could be the result of a combination of these potential changes. As a result of inhibiting IDO induction, sacubitril/valsartan partially reversed tryptophan and kynurenine elevations. The unexpected tryptophan elevation cannot be explained, except by TDO inhibition. There is currently no clear understanding of sacubitril/valsartan's potential effects on tryptophan metabolism, it is a distinct possibility. In addition, valsartan is 95% bound to albumin and may very well displace tryptophan from binding sites which could lead to increased entry of free tryptophan into cardiac and other tissues thereby stimulating its flux through the kynurenine pathway[46]. A lack of more information on the impact of immune disturbances in CHF on tryptophan metabolism along the kynurenine pathway, indicating a need for further research of the role of tryptophan metabolism in the pathophysiology and therapy of the disease.

Our experimental results indicate that sacubitril/valsartan inhibits IDO in myocardial tissue and it inhibits the metabolism of tryptophan to kynurenine, as well as inflammatory. Surprisingly, tryptophan/kynurenine metabolism is related to the inflammatory response[47, 48]. In summary, sacubitril/valsartan may modulate the cross-talk between tryptophan/kynurenine metabolism and the inflammatory response in vivo **(Fig.6)**. On the basis of the original inhibition of RAAS and the activation of NPs, sacubitril/valsartan can also regulate the metabolism of CHF rats in vivo, and it can have a therapeutic effect of 1+1>2.

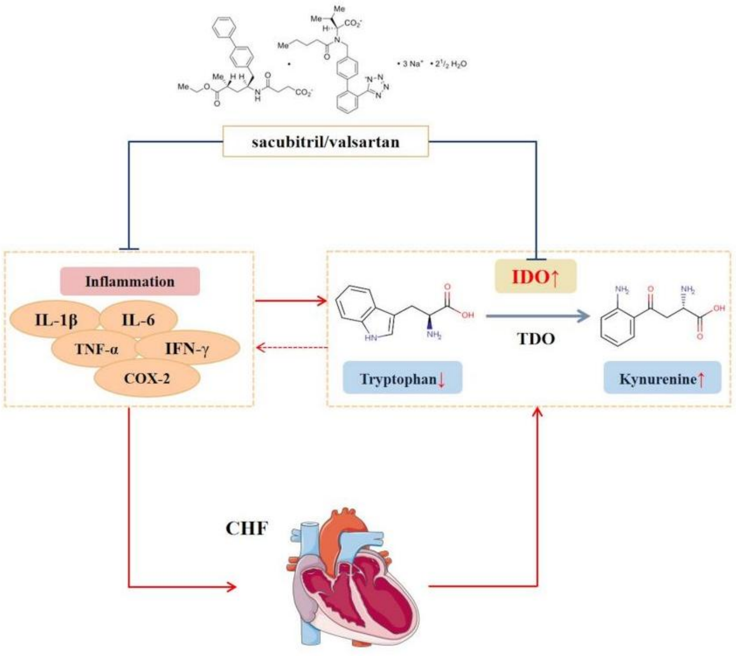

Figure 6 Sacubitril/valsartan regulating tryptophan/kynurenine metabolism and inhibiting inflammation in the treatment of CHF

5 Conclusion

Sacubitril/valsartan can ameliorate cardiac function and ventricular remodeling in CHF rats, at least in part through inhibiting the tryptophan/kynurenine metabolism and inflammation.

6 Acknowledgments

We would like to thank Lin Sun and Zhen Yang for the technical support. Thanks to the National Natural Science Foundation of China (No.81774071) and the Tianjin Natural Science Foundation (No.21JCQNJC01170).

7 Authors Contributions

All authors contributed to the study conception and design. Material preparation, data collection and analysis were performed by Yuli Wang, Yun Deng, and Jiaqi Zhang, Shuangcui Wang, and Xijuan Jiang. The first draft of the manuscript was written by Jiali Gan and all authors commented on previous versions of the manuscript. All authors read and approved the final manuscript.

8 Data availability statement

Correspondence and requests for materials should be addressed to 17320291675@163.com.

9 Competing Interests Statement

The authors have no relevant financial or non-financial interests to disclose.

10 Funding

This work was supported by the National Natural Science Foundation of China (Grant No.81774071) and the Tianjin Natural Science Foundation (Grant No.21JCQNJC01170).

11 Ethics approval

All methods and procedures for the study were performed in accordance with ARRIVE guidelines. Approval was granted by the Ethics Committee of Tianjin University of Traditional Chinese Medicine Ethics Committee on Laboratory Animal (No.TCM-LAEC2020101). All methods were carried out in accordance with relevant guidelines and regulations.

References:

[1] Tsao CW, Aday AW, Almarzooq ZI, Alonso A, Beaton AZ, Bittencourt MS, et al. Heart Disease and Stroke Statistics-2022 Update: A Report From the American Heart Association. CIRCULATION. 2022 2022-02-22;145(8):e153-639.

[2] Ziaeian B, Fonarow GC. Epidemiology and aetiology of heart failure. NAT REV CARDIOL. 2016 2016-06-01;13(6):368-78.

[3] Baman JR, Ahmad FS. Heart Failure. JAMA-J AM MED ASSOC. 2020 2020-09-08;324(10):1015.

[4] Suematsu Y, Jing W, Nunes A, Kashyap ML, Khazaeli M, Vaziri ND, et al. LCZ696 (Sacubitril/Valsartan), an Angiotensin-Receptor Neprilysin Inhibitor, Attenuates Cardiac Hypertrophy, Fibrosis, and Vasculopathy in a Rat Model of Chronic Kidney Disease. J CARD FAIL. 2018 2018-04-01;24(4):266-75.

[5] Solomon SD, McMurray J, Anand IS, Ge J, Lam C, Maggioni AP, et al. Angiotensin-Neprilysin Inhibition in Heart Failure with Preserved Ejection Fraction. NEW ENGL J MED. 2019 2019-10-24;381(17):1609-20.

[6] Desai AS, Vaduganathan M, Cleland JG, Claggett BL, Barkoudah E, Finn P, et al. Mode of Death in Patients With Heart Failure and Preserved Ejection Fraction: Insights From PARAGON-HF Trial. CIRC-HEART FAIL. 2021 2021-12-01;14(12):e8597.

[7] Khder Y, Shi V, McMurray J, Lefkowitz MP. Sacubitril/Valsartan (LCZ696) in Heart Failure. Handb Exp Pharmacol. 2017 2017-01-20;243:133-65.

[8] Schiattarella GG, Rodolico D, Hill JA. Metabolic inflammation in heart failure with preserved ejection fraction. CARDIOVASC RES. 2021 2021-01-21;117(2):423-34.

[9] McGarrah RW, Crown SB, Zhang GF, Shah SH, Newgard CB. Cardiovascular Metabolomics. CIRC RES. 2018 2018-04-27;122(9):1238-58.

[10] Ussher JR, Elmariah S, Gerszten RE, Dyck JR. The Emerging Role of Metabolomics in the Diagnosis and Prognosis of Cardiovascular Disease. J AM COLL CARDIOL. 2016 2016-12-27;68(25):2850-70.

[11] Wu T, Yao H, Zhang B, Zhou S, Hou P, Chen K. kappa Opioid Receptor Agonist Inhibits Myocardial Injury in Heart Failure Rats through Activating Nrf2/HO-1 Pathway and Regulating Ca(2+)-SERCA2a. OXID MED CELL LONGEV. 2021 2021-01-20;2021:7328437.

[12] Cuijpers I, Carai P, Mendes-Ferreira P, Simmonds SJ, Mulder P, Miranda-Silva D, et al. The effect of different anaesthetics on echocardiographic evaluation of diastolic dysfunction in a heart failure with preserved ejection fraction model. SCI REP-UK. 2020 2020-09-24;10(1):15701.

[13] Chen J, Chemaly ER, Liang LF, LaRocca TJ, Yaniz-Galende E, Hajjar RJ. A new model of congestive heart failure in rats. AM J PHYSIOL-HEART C. 2011 2011-09-01;301(3):H994-1003.

[14] Kuwahara K. The natriuretic peptide system in heart failure: Diagnostic and therapeutic implications. PHARMACOL THERAPEUT. 2021 2021-11-01;227:107863.

[15] Gu J, Noe A, Chandra P, Al-Fayoumi S, Ligueros-Saylan M, Sarangapani R, et al. Pharmacokinetics and pharmacodynamics of LCZ696, a novel dual-acting angiotensin receptor-neprilysin inhibitor (ARNi). J CLIN PHARMACOL. 2010 2010-04-01;50(4):401-14.

[16] Dargad RR, Prajapati MR, Dargad RR, Parekh JD. Sacubitril/valsartan: A novel angiotensin receptor-neprilysin inhibitor. INDIAN HEART J. 2018 2018-07-01;70 Suppl 1(Suppl 1):S102-10.

[17] Ayalasomayajula S, Langenickel T, Pal P, Boggarapu S, Sunkara G. Clinical Pharmacokinetics of Sacubitril/Valsartan (LCZ696): A Novel Angiotensin Receptor-Neprilysin Inhibitor. CLIN PHARMACOKINET. 2017 2017-12-01;56(12):1461-78.

[18] Fabris E, Merlo M, Rapezzi C, Ferrari R, Metra M, Frigerio M, et al. Sacubitril/Valsartan: Updates and Clinical Evidence for a Disease-Modifying Approach. DRUGS. 2019 2019-09-01;79(14):1543-56.

[19] Rubattu S, Triposkiadis F. Resetting the neurohormonal balance in heart failure (HF): the relevance of the natriuretic peptide (NP) system to the clinical management of patients with HF. HEART FAIL REV. 2017 2017-05-01;22(3):279-88.

[20] Ishii M, Kaikita K, Sato K, Sueta D, Fujisue K, Arima Y, et al. Cardioprotective Effects of LCZ696 (Sacubitril/Valsartan) After Experimental Acute Myocardial Infarction. JACC-BASIC TRANSL SC. 2017 2017-12-01;2(6):655-68.

[21] Kristensen SL, Preiss D, Jhund PS, Squire I, Cardoso JS, Merkely B, et al. Risk Related to Pre-Diabetes Mellitus and Diabetes Mellitus in Heart Failure With Reduced Ejection Fraction: Insights From Prospective Comparison of ARNI With ACEI to Determine Impact on Global Mortality and Morbidity in Heart Failure Trial. CIRC-HEART FAIL. 2016 2016-01-01;9(1).

[22] Xu Y, Chen Y, Zhao JW, Li C, Wang AY. Effect of Angiotensin-Neprilysin Versus Renin-Angiotensin System Inhibition on Renal Outcomes: A Systematic Review and Meta-Analysis. FRONT PHARMACOL. 2021 2021-01-20;12:604017.

[23] Galo J, Celli D, Colombo R. Effect of Sacubitril/Valsartan on Neurocognitive Function: Current Status and Future Directions. AM J CARDIOVASC DRUG. 2021 2021-05-01;21(3):267-70.

[24] Dindas F, Gungor H, Ekici M, Akokay P, Erhan F, Dogdus M, et al. Angiotensin receptor-neprilysin inhibition by sacubitril/valsartan attenuates doxorubicin-induced cardiotoxicity in a pretreatment mice model by interfering with oxidative stress, inflammation, and Caspase 3 apoptotic pathway. ANATOL J CARDIOL. 2021 2021-11-01;25(11):821-8.

[25] Bell E, Desuki A, Karbach S, Gobel S. Successful treatment of doxorubicin-induced cardiomyopathy with low-dose sacubitril/valsartan: a case report. EUR HEART J-CASE REP. 2022 2022-10-01;6(10):c396.

[26] Quagliariello V, De Laurentiis M, Rea D, Barbieri A, Monti MG, Carbone A, et al. The SGLT-2 inhibitor empagliflozin improves myocardial strain, reduces cardiac fibrosis and pro-inflammatory cytokines in non-diabetic mice treated with doxorubicin. CARDIOVASC DIABETOL. 2021 2021-07-23;20(1):150.

[27] Norre T, Grimm D, Simonsen U. Sacubitril/valsartan, sodium-glucose cotransporter 2 inhibitors and vericiguat for congestive heart failure therapy. BASIC CLIN PHARMACOL. 2022 2022-04-01;130(4):425-38.

[28] Kimball TH, Vondriska TM. Metabolism, Epigenetics, and Causal Inference in Heart Failure. TRENDS ENDOCRIN MET. 2020 2020-03-01;31(3):181-91.

[29] Yamazaki F, Kuroiwa T, Takikawa O, Kido R. Human indolylamine 2,3-dioxygenase. Its tissue distribution, and characterization of the placental enzyme. BIOCHEM J. 1985 1985-09-15;230(3):635-8.

[30] Lob S, Konigsrainer A, Zieker D, Brucher BL, Rammensee HG, Opelz G, et al. IDO1 and IDO2 are expressed in human tumors: levo- but not dextro-1-methyl tryptophan inhibits tryptophan catabolism. CANCER IMMUNOL IMMUN. 2009 2009-01-01;58(1):153-7.

[31] Munn DH, Mellor AL. Indoleamine 2,3 dioxygenase and metabolic control of immune responses. TRENDS IMMUNOL. 2013 2013-03-01;34(3):137-43.

[32] Wongpraparut N, Pengchata P, Piyophirapong S, Panchavinnin P, Pongakasira R, Arechep N, et al. Indoleamine 2,3 dioxygenase (IDO) level as a marker for significant coronary artery disease. BMC CARDIOVASC DISOR. 2021 2021-07-26;21(1):353.

[33] Li M, Kwok MK, Fong S, Schooling CM. Indoleamine 2,3-dioxygenase and ischemic heart disease: a Mendelian Randomization study. SCI REP-UK. 2019 2019-06-11;9(1):8491.

[34] Chon SY, Hassanain HH, Gupta SL. Cooperative role of interferon regulatory factor 1 and p91 (STAT1) response elements in interferon-gamma-inducible expression of human indoleamine 2,3-dioxygenase gene. J BIOL CHEM. 1996 1996-07-19;271(29):17247-52.

[35] Banzola I, Mengus C, Wyler S, Hudolin T, Manzella G, Chiarugi A, et al. Expression of Indoleamine 2,3-Dioxygenase Induced by IFN-gamma and TNF-alpha as Potential Biomarker of Prostate Cancer Progression. FRONT IMMUNOL. 2018 2018-01-20;9:1051.

[36] Garrison AM, Parrott JM, Tunon A, Delgado J, Redus L, O'Connor JC. Kynurenine pathway metabolic balance influences microglia activity: Targeting kynurenine monooxygenase to dampen neuroinflammation. PSYCHONEUROENDOCRINO. 2018 2018-08-01;94:1-10.

[37] Li Z, Zhao H, Wang J. Metabolism and Chronic Inflammation: The Links Between Chronic Heart Failure and Comorbidities. FRONT CARDIOVASC MED. 2021 2021-01-20;8:650278.

[38] Mezzaroma E, Toldo S, Farkas D, Seropian IM, Van Tassell BW, Salloum FN, et al. The inflammasome promotes adverse cardiac remodeling following acute myocardial infarction in the mouse. P NATL ACAD SCI USA. 2011 2011-12-06;108(49):19725-30.

[39] Mangan M, Olhava EJ, Roush WR, Seidel HM, Glick GD, Latz E. Targeting the NLRP3 inflammasome in inflammatory diseases. NAT REV DRUG DISCOV. 2018 2018-08-01;17(8):588-606.

[40] Higashikuni Y, Liu W, Numata G, Tanaka K, Fukuda D, Tanaka Y, et al. NLRP3 Inflammasome Activation Through Heart-Brain Interaction Initiates Cardiac Inflammation and Hypertrophy During Pressure Overload. CIRCULATION. 2023 2023-01-24;147(4):338-55.

[41] Razquin C, Ruiz-Canela M, Toledo E, Hernandez-Alonso P, Clish CB, Guasch-Ferre M, et al. Metabolomics of the tryptophan-kynurenine degradation pathway and risk of atrial fibrillation and heart failure: potential modification effect of Mediterranean diet. AM J CLIN NUTR. 2021 2021-11-08;114(5):1646-54.

[42] Li X, Zhu Q, Wang Q, Zhang Q, Zheng Y, Wang L, et al. Protection of Sacubitril/Valsartan against Pathological Cardiac Remodeling by Inhibiting the NLRP3 Inflammasome after Relief of Pressure Overload in Mice. CARDIOVASC DRUG THER. 2020 2020-10-01;34(5):629-40.

[43] Ortega-Paz L, Cristobal H, Ortiz-Perez JT, Garcia DFP, Mendieta G, Sandoval E, et al. Direct actions of dapagliflozin and interactions with LCZ696 and spironolactone on cardiac fibroblasts of patients with heart failure and reduced ejection fraction. ESC HEART FAIL. 2023 2023-02-01;10(1):453-64.

[44] Servia L, Jove M, Sol J, Pamplona R, Badia M, Montserrat N, et al. A prospective pilot study using metabolomics discloses specific fatty acid, catecholamine and tryptophan metabolic pathways as possible predictors for a negative outcome after severe trauma. SCAND J TRAUMA RESUS. 2019 2019-05-22;27(1):56.

[45] Badawy AA, Guillemin G. The Plasma [Kynurenine]/[Tryptophan] Ratio and Indoleamine 2,3-Dioxygenase: Time for Appraisal. INT J TRYPTOPHAN RES. 2019 2019-01-20;12:519664178.

[46] Smith SA, Pogson CI. The metabolism of L-tryptophan by isolated rat liver cells. Effect of albumin binding and amino acid competition on oxidatin of tryptophan by tryptophan 2,3-dioxygenase. BIOCHEM J. 1980 1980-03-15;186(3):977-86.

[47] Gelpi M, Ueland PM, Troseid M, Mocroft A, Lebech AM, Ullum H, et al. Abdominal Adipose Tissue Is Associated With Alterations in Tryptophan-Kynurenine Metabolism and Markers of Systemic Inflammation in People With Human Immunodeficiency Virus. J INFECT DIS. 2020 2020-01-14;221(3):419-27.

[48] Cervenka I, Agudelo LZ, Ruas JL. Kynurenines: Tryptophan's metabolites in exercise, inflammation, and mental health. SCIENCE. 2017 2017-07-28;357(6349).
