# Supplementary material for: Sacubitril/valsartan ameliorates cardiac function and ventricular remodeling in CHF rats via the inhibition of the tryptophan/kynurenine metabolism and inflammation
Source: Sci Rep. 2024 May 29;14:12377. doi: 10.1038/s41598-024-62472-7 (PMC11136956; doi:10.1038/s41598-024-62472-7)
Supplement: Supplementary file 2 — Supplementary Information 2. [file 41598_2024_62472_MOESM2_ESM.pdf]

Supplementary Material

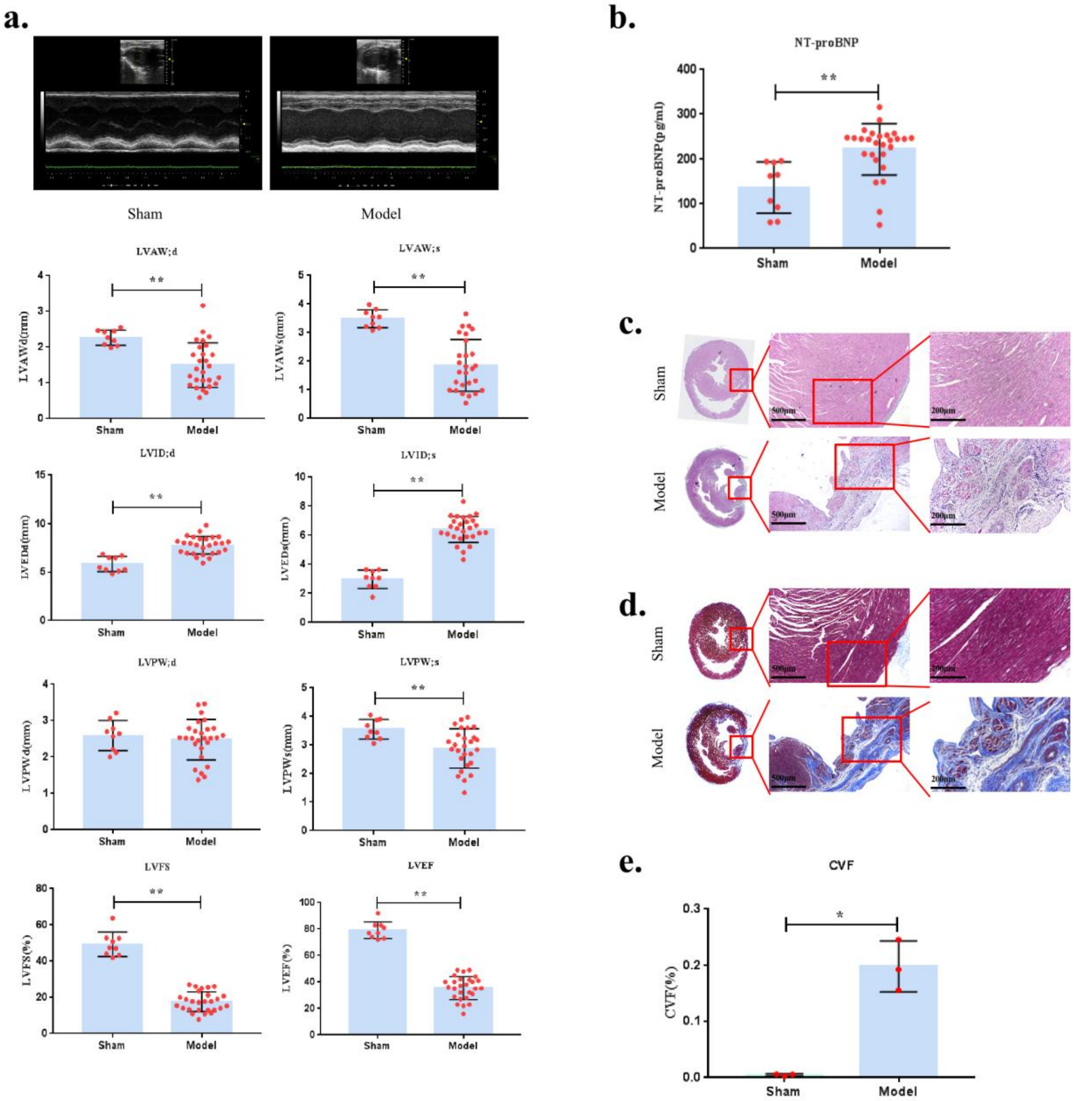

Supplementary Figure 1　A rat model of post-myocardial infarction chronic heart failure via left anterior descending and exhaustive swimming

(a) M-mode echocardiographyand cardiac function parameters.　LVAW;d, left ventricular anterior wall thickness of diastolic.　LVAW;s, left ventricular anterior wall of systolic.　LVID;s, left ventricular internal diameter of systole.　LVID;d, left ventricular internal diameter of diastolic. LVPW;d, left ventricular posterior wall of diastolic.　LVPW;s, left ventricular posterior wall of systolic.　LVEF, left ventricular ejection fraction.　LVFS, left ventricular percent fractional shortening.　**(b)** NT-proBNP in serum.　NT-proBNP, N terminal pro Brain natriuretic peptidepeptide.　**(c)** HE stain image.　**(d)** Masson stain image.　**(e)** CVF.　CVF, collagen volume fraction.　Compaerd with Sham group, $^{**}P < 0.01$,$^{*}P < 0.05$.

	Sham（n=9）	Model（n=27）
LVAW;d（mm）	2.27±0.21	1.50±0.63**
LVAW;s（mm）	3.49±0.31	1.85±0.90**
LVID;d（mm）	5.83±0.78	7.76±0.92**
LVID;s（mm）	2.97±0.63	6.40±0.89**
LVPW;d（mm）	2.59±0.41	2.47±0.56
LVPW;s（mm）	3.55±0.44	2.88±0.69**
LVEF（%）	79.11±6.35	35.51±8.68**
LVFS（%）	49.32±6.74	17.60±5.43**
NT-proBNP(pg/ml)	136.69±57.50	221.64±57.10**
CVF(%)	0.47±0.19	17.13*

Supplementary Tabel 1 Cardiac Function Parameters、NT-proBNP、CVF

Compaerd with Sham group, $^{**}P < 0.01$,$^{*}P < 0.05$.

Echocardiography, NT-proBNP, and histological Analysis of Cardiac Tissue were performed After exhaustive swimming 28 days to identificate CHF model. Our experiment results indicate that, compared with Sham group, LVAW;d, LVAW;s, LVPW;S, LVEF, and LVFS of CHF model group were significantly decreased (P <0.01), and LVID;s and LVID;d increased significantly (P<0.01) **(Fig.S.1a and Tab.S.2).** NT-proBNP was remarkably higher with CHF model group versus Sham group(P<0.01) **(Fig.S.1b and Tab.S.2)**. HE **(Fig.S.1c)** and Masson **(Fig.S.1d)** staining of the cardiac tissue section also indicated that the myocardial injury of CHF rats was serious, and the myocardial fibrosis was obvious. And masson staining showed that collagen volume fraction(CVF) of CHF model rtas was significantly higher (P<0.05) **(Fig.S.1e and Tab.S.2)**. The above is consistent with chronic heart failure. Therefore , A stable and successful rat model of post-myocardial infarction chronic heart failure via left anterior descending and exhaustive swimming.

	Sham	Model	S
LVAW;d（mm）	2.40±0.23	1.87±0.61*	2.24±0.67
LVAW;s（mm）	3.66±0.35	2.34±0.85**	3.00±1.10
LVID;d（mm）	5.80±0.79	7.96±1.22**	7.17±1.02
LVID;s（mm）	3.21±0.54	6.53±1.24**	5.12±1.22#
LVPW;d（mm）	2.81±0.82	2.54±0.34	2.76±0.51
LVPW;s（mm）	3.66±0.66	3.04±0.58*	3.37±0.57
LVEF（%）	75.09±6.12	36.76±6.11**	54.36±14.77#
LVFS（%）	44.50±7.08	18.31±4.99**	29.18±10.38#
HW/BW	0.37±0.04	0.44±0.06*	0.42±0.12
NT-proBNP（pg/ml）	310.20±78.34	468.79±85.88**	293.99±63.56##
CVF（%）	0.27±0.12	16.14±1.05**	11.01±3.69#

Supplementary Tabel 2 Cardiac Function Parameters、HW/BW、NT-proBNP、CVF

HW/BW, Heart weight/Body weight. Compared with Sham group, $^{**}P$<0.01, $^{**}P$<0.05, compared with Model group, $^{\#\#}P$<0.01, $^{\#}P$<0.05.

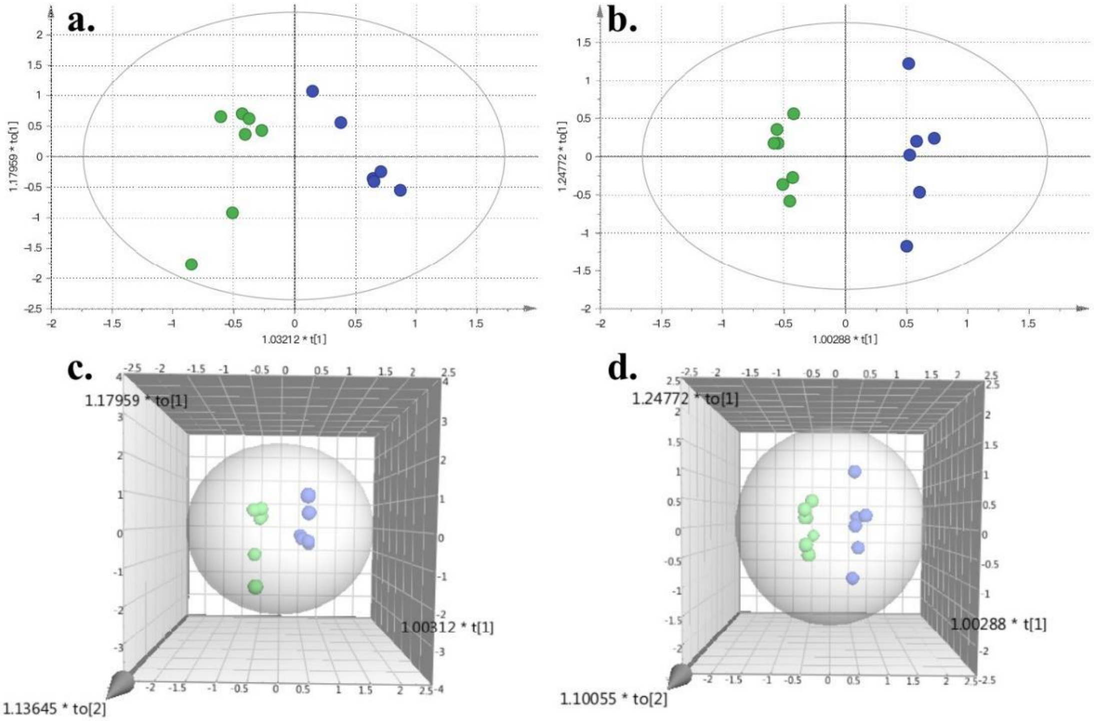

Supplementary Figure 2 Serum Metabolic Profiles Analysis
(a) OPLS-DA(ESI+) **(b)** OPLS-DA(ESI-) **(c)** OPLS-DA 3D (ESI+) **(d)** OPLS-DA 3D (ESI-)

Model	Type	A	N	R^2X(cum)	R^2Y(cum)	Q^2(cum)
Positive Mode	PCA-X	3	20	0.484	-	0.213
Negative Mode	PCA-X	3	20	0.479	-	0.185
Positive Mode	PLS-DA	3	20	0.467	0.859	0.433
Negative Mode	PLS-DA	3	20	0.446	0.889	0.538

Supplementary Tabel 3 Mathematical model parameters of sham group, model group, and S group

R^2X is used to evaluate the validity of the model, R^2Y represents the interpretation rate of the model, Q^2 represents the prediction ability of the model. The closer the parameter is to 1, the higher the interpretation rate is. R^2 and Q^2 are greater than 0.9, indicating that the model has excellent explanatory and predictive ability, and greater than 0.5 is considered to be reliable.

No.	Name	Total	Expected	Hits	Raw p	Holm p	FDR	-log10(p)	Impact
**1**	**Tryptophan metabolism**	**41**	**0.21736**	**2**	**0.018187**	**1**	**1**	**1.7402**	**0.23722**
2	Glycine, serine and threonine metabolism	34	0.18025	1	0.16702	1	1	0.77724	0.02408
3	Glycerophospholipid metabolism	36	0.19085	1	0.17603	1	1	0.75441	0.01736
4	Valine, leucine and isoleucine biosynthesis	8	0.042412	1	0.041729	1	1	1.3796	0
5	Aminoacyl-tRNA biosynthesis	48	0.25447	2	0.024554	1	1	1.6099	0

Supplementary Tabel 4 Metabolic pathway analysis results

Total: the total number of compounds in the channel. Hits: the actual matching value of the data uploaded by the user. Rawp: calculate the original P value; Holm p: adjust the P value by Holm-Bonferoni method. FDR p: use the false discovery rate Adjusted P value. Impact: Path impact value calculated by path topology analysis.

	IDO	GAPDH
Repeat 1	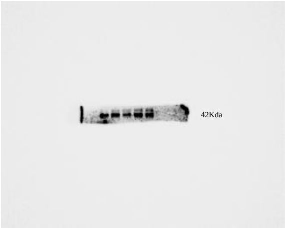	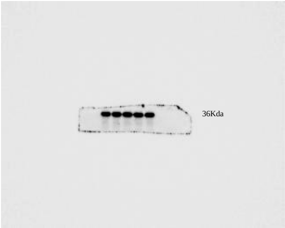
Repeat 2	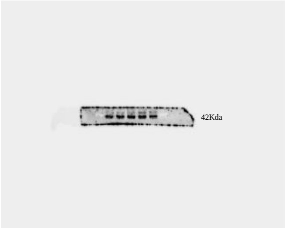	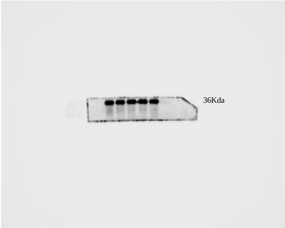
Repeat 3	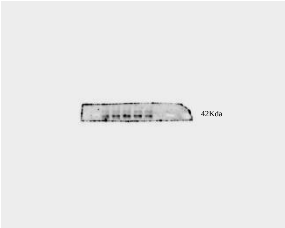	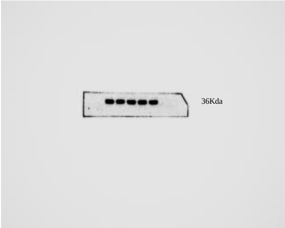
Repeat 4	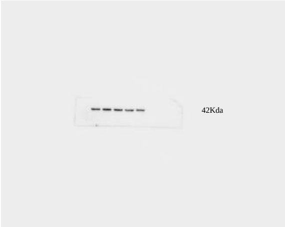	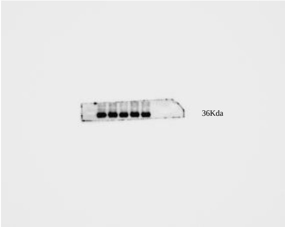
Repeat 5	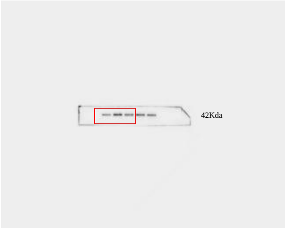	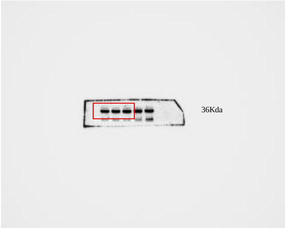

Supplementary Tabel 5 The original western blot images for five repeats

The groups of western blot are in order: sham group, model group, sacubitril/valsartan group, Shengmai Yin group, and Shengmai Yin + sacubitril/valsartan group.
